# Land Snails as a Diet Diversification Proxy during the Early Upper Palaeolithic in Europe

**DOI:** 10.1371/journal.pone.0104898

**Published:** 2014-08-20

**Authors:** Javier Fernández-López de Pablo, Ernestina Badal, Carlos Ferrer García, Alberto Martínez-Ortí, Alfred Sanchis Serra

**Affiliations:** 1 Institut Català de Paleoecologia Humana i Evolució Social, Zona Educacional 4 Campus Sescelades (Edifici W3), Tarragona, Spain; 2 Àrea de Prehistòria, Universitat Rovira i Virgili (URV), Tarragona, Spain; 3 Departament de Prehistòria i Arqueologia, Facultat de Geografia i Història, Universitat de València, València, Spain; 4 Museu de Prehistòria de València, SIP (Servei d'Investigació Prehistòrica), Diputació de València, València, Spain; 5 Museu Valencià d'Història Natural & i\ Biotaxa, Valencia, Spain; Universidade do Algarve, Portugal

## Abstract

Despite the ubiquity of terrestrial gastropods in the Late Pleistocene and Holocene archaeological record, it is still unknown when and how this type of invertebrate resource was incorporated into human diets. In this paper, we report the oldest evidence of land snail exploitation as a food resource in Europe dated to 31.3-26.9 ka yr cal BP from the recently discovered site of Cova de la Barriada (eastern Iberian Peninsula). Mono-specific accumulations of large *Iberus alonensis* land snails (Ferussac 1821) were found in three different archaeological levels in association with combustion structures, along with lithic and faunal assemblages. Using a new analytical protocol based on taphonomic, microX-Ray Diffractometer (DXR) and biometric analyses, we investigated the patterns of selection, consumption and accumulation of land snails at the site. The results display a strong mono-specific gathering of adult individuals, most of them older than 55 weeks, which were roasted in ambers of pine and juniper under 375°C. This case study uncovers new patterns of invertebrate exploitation during the Gravettian in southwestern Europe without known precedents in the Middle Palaeolithic nor the Aurignacian. In the Mediterranean context, such an early occurrence contrasts with the neighbouring areas of Morocco, France, Italy and the Balkans, where the systematic nutritional use of land snails appears approximately 10,000 years later during the Iberomaurisian and the Late Epigravettian. The appearance of this new subsistence activity in the eastern and southern regions of Spain was coeval to other demographically driven transformations in the archaeological record, suggesting different chronological patterns of resource intensification and diet broadening along the Upper Palaeolithic in the Mediterranean basin.

## Introduction

Diet change is a widely debated research topic of the Middle to Upper Palaeolithic transition. Studies on vertebrate prey mobility, size and body biomass suggest that, in many areas of Europe, the first anatomically modern humans (AMH) had a broader diet than Neanderthals, who mainly focused on large- and medium-size herbivores [1]–[3]. However, this view has been called into a question by the increasing body of archaeological evidence indicating that Neanderthals' subsistence also relied on a varied range of resources including plants, fish, birds, shellfish, tortoises, marine mammals and rabbits [4]–[15]. In this context, terrestrial molluscs were not believed to have been of any importance in the study of the dietary change and nutritional ecology during the Middle to Upper Palaeolithic transition. Unlike the increasing evidence for the consumption of marine molluscs amongst the Neanderthals, there is a no clear signal of land snail exploitation during the Middle Palaeolithic, where terrestrial molluscs are considered intrusive in archaeological contexts, accumulated by thanatocoenoses or transported by non-human predators. Furthermore, the use of land snails as a food resource has been openly questioned in several Early Upper Palaeolithic contexts on the grounds of taphonomic and spatial analyses [16]. However, as posed by Lubell [17]–[18], the environmental interest of Late Pleistocene land snails and the paucity of specific studies focused on taxonomic, taphonomic, quantitative and biometric studies have prevented an understanding of the beginning, context and specific modalities of this type of subsistence activity.

In this paper, we report new evidence of land snail consumption from the Gravettian archaeological site of Cova de la Barriada (Benidorm, Spain) in the southeastern Iberian Peninsula, dated to 31.3–26.9 yr cal BP. This site has yielded mono-specific concentrations of large *Iberus alonensis* (Ferussac 1821) land snails associated with occupational features, lithic artefacts and mammalian faunal assemblages accumulated by humans. Through taphonomic and biometric analyses, we will investigate the patterns of selection and cultural accumulation of this species of edible land snail at the site. This case study illustrates new patterns of economic diversification during the Early Upper Palaeolithic in the eastern and southern Iberian Peninsula, which is not documented in other European and circum-Mediterranean regions until the Late Upper Palaeolithic, more than 10,000 years later.

## Materials and Methods

### 1. Site description

**Figure 1:**
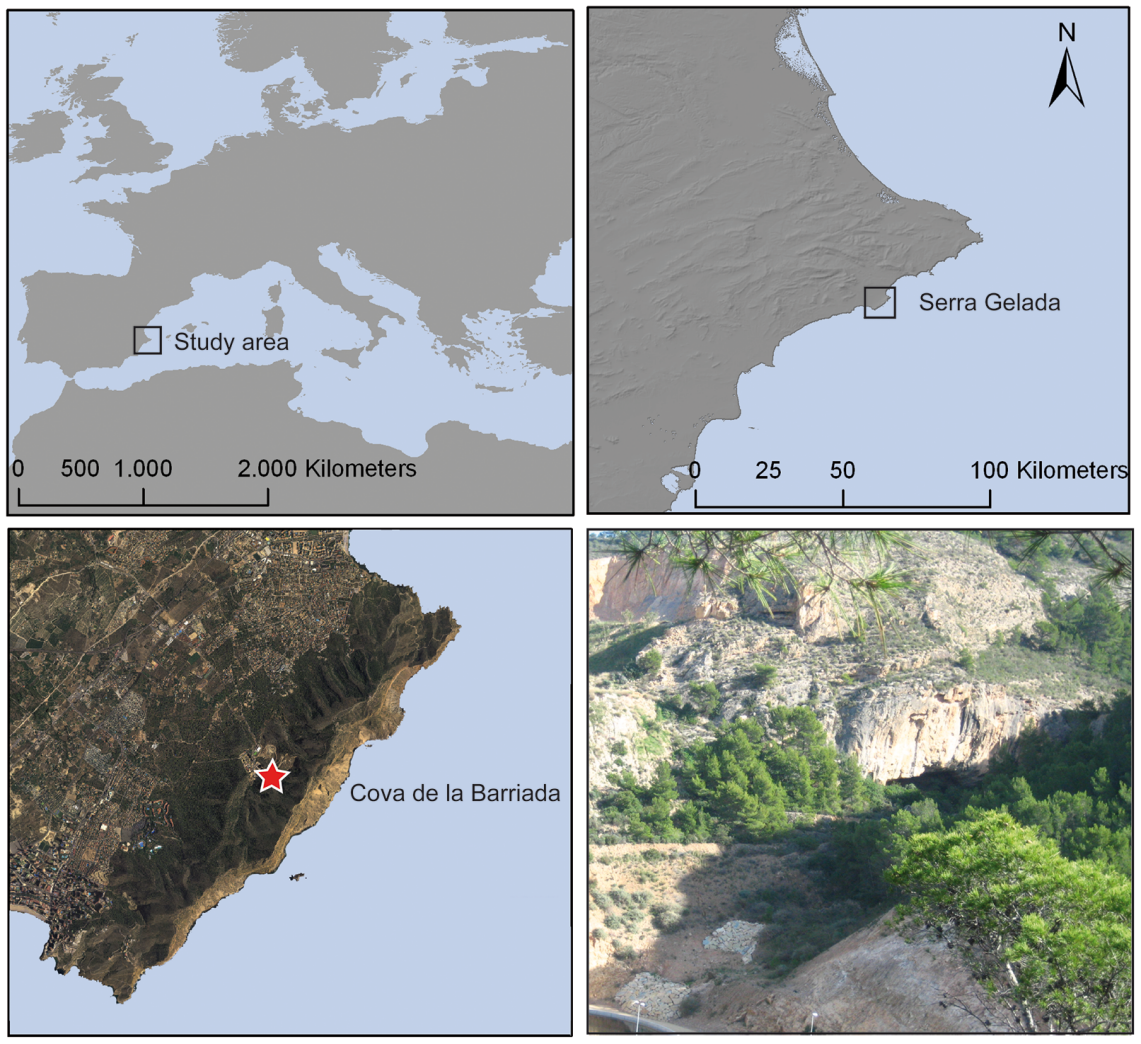
Study area and site location. Bottom right: general view of Cova de la Barriada site.

Cova de la Barriada is formed by two connected rockshelters, so-called lower and upper rockshelters, respectively, located at the base of a tectonic escarpment of Mesozoic limestone on the western slope of the Serra Gelada mountain (Figure 1). The site is oriented towards the NW at 180 m.a.s.l.

Excavation was undertaken in January 2011 and consisted of a series of three test pits to evaluate the preservation of the sedimentary fill. Archaeological Fieldwork permit (Ref.2010/1023-A) was issued by the Dirección General de Patrimonio (Generalitat Valenciana, Valencia, Spain) to Javier Fernández-López de Pablo (Permit number: 2010/1023-A). In the lower rockshelter, Test Pit 2, which is 2 m^2^, revealed a thin remnant Holocene deposit overlying the limestone bedrock. Despite the lack of lithic and faunal assemblages, two different anthropic accumulations of land snails (*Sphincterochila candidissima*) and marine molluscs (*Cerastoderma glaucum* and *Patella* sp) were documented in this deposit, suggesting an Early to Middle Holocene age (likely Mesolithic).

In the upper rockshelter, the archaeological fill was formed by colluvial sedimentation that, for the most part, has been eroded away by water (karstic) erosion and subsequent transport as well as recent husbandry and looting activities. Test Pit 3, which is 1 m^2^, yielded archaeological materials in clearly secondary position from the dismantled Late Pleistocene deposits.

**Figure 2:**
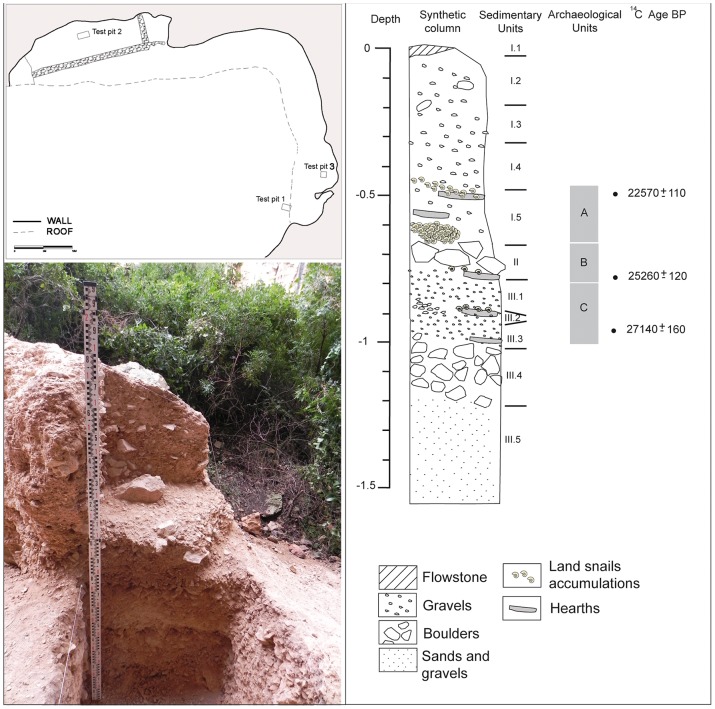
Up left: plan of Cova de la Barriada site with the location of test pits. Bottom left: Test pit 1, once excavated. Right: Synthetic column of the test pit 1 stratigraphy.

Only Test Pit 1, located at the south of the upper rockshelter, right outside of the roofed area, preserved *in situ* archaeological levels with combustion structures and associated lithic, faunal and land snail assemblages. The archaeo-sedimentary deposit is formed by a succession of three main stratigraphic units, subdivided into several subunits with different contents of boulders, angular blocks and gravels in a matrix of sands and silts (Figure 2). From top to bottom, the stratigraphic sequence is described as follows:

Unit I is composed of five subunits. Subunit I.1 is a calcareous flowstone of horizontal geometry. Subunits I.2 to I.5 are mainly composed by very pale brown (10 YR 7/3 and 7/4) thin fraction (70%) and angular heterometric gravels. Subunit I.5, which overlies Unit II with an angular unconformity, yielded archaeological evidence (artefacts and features) that correspond to Archaeological Unit A.

Unit II is a 10-cm thick deposit of massive structure containing a heterometric thick fraction of gravitational boulders and angular blocks and gravels in a matrix of very pale brown (10 YR 8/3) thin fraction. It dips E-W with an inclination of 12–10°. This stratigraphic unit corresponds to Archaeological Unit B, containing both artefacts and features.

Unit III is composed of five different subunits. Subunits III.1 and III.3 contain 90% of greyish orange (10 YR 7/4) sands and silts partially separated by a laterally discontinuous subunit of gravels (III.2). The underlying subunit III.4 (10 YR 8/3 and 8/4) is predominantly composed of a thick fraction (boulders and angular blocks), whereas subunit III.5 is mainly formed by massive structure sands and silts. Occupational evidence (Archaeological Unit C) is restricted to subunits III.1 and III.3.

Archaeological units A, B and C have yielded Early Upper Palaeolithic artefacts, faunal assemblages and combustion structures whose basic morphological and dimensional attributes are presented in Table 1. Despite the partial conservation of the combustion structures, most of them (EC-1, EC-2, EC-3, EC-4 and EC-5) have a flat section associated with heterogeneous carbonaceous lenses and fire-cracked limestone blocks. In contrast, combustion structure BM, which was partially documented because it extended outside the limits of the test pit, has a shallow pit morphology and a concave section containing homogeneous carbonaceous sediments with abundant charcoal. On the other hand, combustion structure EC-6 has an irregular concave section associated with burnt and fire-cracked limestone blocks.

**Table 1:** Descriptive attributes of the combustion structures documented in test pit 1.

Combustion structures	Sedimentary	Level	Section	Area	Slope	^14^C Age BP
	Sub-unit			conserved*(m2)	(degrees)	
EC-1	I.5	A	Flat	0.1644	11	22750±110
EC-2	I.5	A	Flat	0.0843	7	
BM	I.5	A	Concave	0.0805	7	
EC-3	II	B	Flat	0.0997	12	
EC-5	II	B	Flat	0.0320	5	25260±120
EC-4	III.1	C	Flat	0.8669	5	
EC-6	III.3	C	Concave	0.1186	4	27140±160

*Area conserved denotes the surface of the carbonaceous area.

A series of AMS radiocarbon dates from individual and taxonomically determined charcoal samples recovered from the combustion structures were produced to assess the chronology of the stratigraphic sequence (Table 2). The samples are charcoal of *Pinus nigra* from EC-1 level A, Fabaceae charcoal from EC-5 level B and *Juniperus* sp. charcoal from EC-6 level C. All samples were plotted at the time of excavation and analysed at the Beta Analytic Laboratories in London. Calibration was performed using Oxcal v.4.1.3 [19] and the Intcal13 calibration curve [20].

**Table 2:** Radiocarbon dates of the test pit 1 of Cova de la Barriada calibrated with Oxcal 4.2 [19] and the Intcal 13 calibration curve [20].

Level	Context	Sample	Ref.Lab	C^12/13^	^14^C Age BP	2 Sigma Cal BP
A	EC-1	*Pinus nigra*	Beta-296222	−21.9‰	22750±110	27398–26712
B	EC-2	Fabaceae	Beta-296223	−22.5‰	25260±120	29642–28958
C	EC-3	*Juniperus* sp.	Beta-362534	−20.5‰	27140±160	31342–30897

Radiocarbon dates from levels A, B and C yielded significantly different chronologies in accordance with their stratigraphic position, suggesting two hiatuses between archaeological units C and B and archaeological units B and A. The chronological gaps between the above-mentioned levels are consistent with the erosive contact documented between subunits I.A and II and subunits III.1 and III.3.

**Figure 3:**
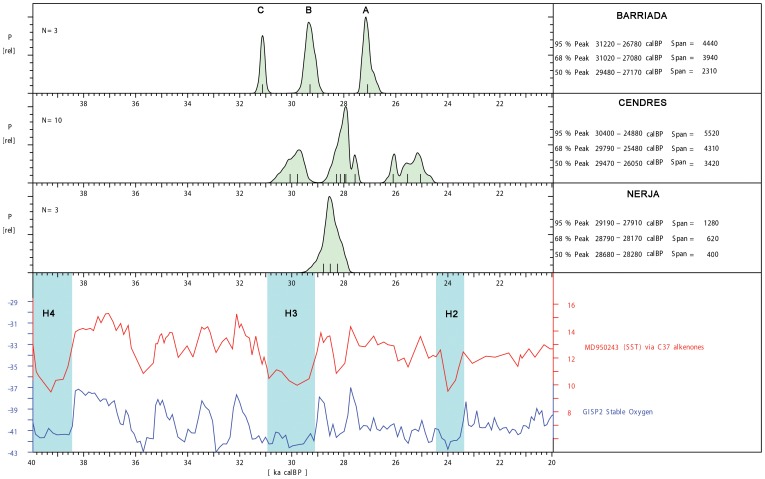
Top: Cumulative calibrated dating probability of the radiocarbon dates from the levels A and B of Cova de la Barriada and the Gravettian units of Nerja [21] and Cendres sites [22] plotted with CalPal (vers. October 2013) [89] using the IntCal13 calibration curve [20]. Bottom: δ^18^O variation from the GISP2 curve [23] and Sea Surface Temperatures obtained from alkenonne data from the Alborán Sea [24].

Figure 3 represents a correlation between the radiocarbon chronology of Levels A and B of Cova de la Barriada and the AMS radiocarbon dates from the well-known Gravettian units of the Nerja and Cendres caves, the regional reference sequences in the Iberian Mediterranean region for this period [21]–[22]. In addition, we compared the summed probability chronological distributions with the global climatic ^18^O GISP2 curve [23] and the regional variations of sea surface temperatures obtained in the Alborán Sea [24]. According to this tentative correlation, Level B falls within the accepted chronology of the H3 Heinrich event in the Mediterranean Sea, whereas Level A appears to fall in Greenland Interstadial 3.

At the time of the Early Upper Palaeolithic occupations in Cova de la Barriada (30–25 ka), the Mediterranean sea level was 90–100 m lower than today, implying a distance of approximately 20 km between the site and the shore line on the basis of local data on marine floor topography [25].

The archaeological and paleontological information presented in this paper involved the direct analysis of 321 paleobotanical specimens, 489 vertebrate specimens and 1484 invertebrate (land snails) specimens.

### 2. Contextual information

A set of Upper Palaeolithic artefacts were recovered in association with the land snails and faunal assemblages in Test Pit 1. Despite the paucity of the lithic assemblages (n = 39), two dihedral-deviated burins were documented at Level A, and two splintered pieces were documented in Levels A and B. In addition, three small umbo-pierced marine shells of *Glycimeris* sp. were recovered in Levels A (n = 2) and C (n = 1).

The palaeobotanical evidence recovered comprises 328 charcoal remains of 9 different taxa (Table 3). They represent a genuine western Mediterranean Late Pleistocene landscape, dominated by cryophilous pines (*Pinus nigra and/or Pinus sylvestris*) and junipers (*Juniperus* sp.), which, in turn, are the most common taxa documented as fuel in the Upper Palaeolithic and other woody plants such as rosemary (*Rosmarinus officinalis*), *Cistus* sp. Fabaceae and Lamiaceae [26]–[28]. In summary, the taxonomic association identified throughout the archaeological sequence indicates a cold ecology with the punctual presence of genuine Mediterranean flora such as the rosemary in level A. The present-day distribution of the *Pinus nigra* and *Pinus sylvestris* forests are found at 1500 m.a.s.l., from medium up to high altitudinal mountain ranges, associated with mean annual precipitation regimes of 500–1000 mm and temperatures between 8 and 13 °C. According to these parameters, we could suggest similar palaeoenvironmental conditions during the Gravettian occupation of the site, as it is also supported by the more complete antracological sequence of Cova de les Cendres, located just 25 km away northward [26].

**Table 3:** Charcoal assemblages recovered in the Gravettian levels of Cova de la Barriada.

	Level A		BM		Level B		Level C	
	n	%	n	%	n	%	n	%
*Pinus nigra*	83	61.02	27	56.25	64	90.14	2	3.03
*Juniperus* sp.	28	20.59	19	39.58	4	5.63	64	96.97
*Rosmarinus officinalis*	2	1.47						
Fabaceae	13	9.56			1	1.41		
Lamiaceae	1	0.74			2	2.82		
*Cistus* sp.			1	2.08				
*Conífera*	5	3.68	1	2.08				
Bark	4	2.94						
Total	136	100	48	100	71	100	66	100

The spectrum of taxa represented in the hearth structure (BM and EC-6) is very narrow: four amongst the 48 charcoal fragments recovered in BM and just two from the 66 charcoal fragments in fireplace EC-6. This might be explained by both the low number of charcoals from each fireplace and their short-term accumulation as a fuel of the last burning episode, thus, reflecting a punctual harvest of firewood. In any case, the flora identified in the combustion structures is in ecological agreement with that recovered from the archaeological units as a result of a longer accumulation process. Both charcoals from fireplaces and archaeological units are the result of human agency.

The faunal assemblage of Test Pit 1 is composed of 489 elements. The ratio of identified specimens at the taxonomic level (NISP) is low, varying between 14.6% in Level A and 2.7% in Level C. Four species of ungulates have been identified: aurochs (*Bos primigenius*), horse (*Equus ferus*), red deer (*Cervus elaphus*) and the Spanish ibex (*Capra pyrenaica*). In addition, lagomorphs are represented throughout the archaeological sequence.

The taphonomic analysis points to both anthropogenic accumulation and post-depositional breakage, with a very marginal contribution by non-human predators. Despite the lack of cut marks, partially explained by the high occurrence of post-depositional calcareous concretions, percussion marks have been clearly identified on diaphyseal fragments of ibex (1), medium-size (2) and large-size (1) ungulates in Levels A and B, as well as a human bite mark on a leporid bone (Table 4).

**Table 4:** Faunal assemblages (NISP) from the test pit 1 of Cova de la Barriada.

	Level A		BM		Level B		Level C	
	n	%	n	%	n	%	n	%
*Bos primigenius*		0					1	0.90
*Capra pyrenaica*	1	0.54			3	1.05		
*Cervus elaphus*	2	1.08			3	1.05		
*Equus ferus*					1	0.39		
Leporidae	24	12.97	3	33.33	17	5.98	2	1.80
Total det	27	14.59	3	33.33	24	8.54	3	2.70
Large size	2	1.08			3	1.05	3	2.70
Medium size	83	44.86			225	79.22	104	93.69
Small size	57	30.81	6	66.66	21	7.39	1	0.90
Undetermined	16	8.64			11	3.87		
Total undet	158	85.41	6	66.66	257	90.49	108	97.29
Total	185	100	9	100	284	100	111	100

Digestive corrosion and alterations indicating the intervention of non-human predators are marginal (just one leporid fragment), suggesting an anthropogenic origin for almost all the osteological remains. Despite the small size of the zooarchaeological dataset, the spectrum of identified species, including red deer, Spanish ibex, horse and aurochs, is consistent with the representation pattern found in other Mediterranean Gravettian contexts such as Levels XIV and XVI of Cova de les Cendres, located just 25 km to the north on the modern coast line [27]. These ungulates suggest the human exploitation of different ecotones such as littoral plains, open forests and abrupt-rocky slopes. On the other hand, the representation of lagomorphs at the site fits with the robust body of datasets indicating that the rabbit is the most abundant taxa found in the Iberian Mediterranean Region throughout the Upper Palaeolithic, especially from the Gravettian onwards [29]–[30].

### 3. Methods

#### 3.1. Taxonomic identification

The land snails analysed in this study −1484 number of identified specimens (NISP) and 832 number of minimal individuals(MNI)- were recovered during the excavations of Test Pit 1, using dry sieving through 5 and 1 mm mesh. Archaeological land snail specimens were examined and compared with published materials in the regional taxonomic literature [31], [32] and reference gastropod collections at the Natural History Museum of Valencia and the Department of Zoology at the University of Valencia. The quantification procedures were based on the NISP (excluding the whorl fragments smaller than 3 mm) with the MNI based on apical counts [33]. The land snails and archaeological materials presented in this study are deposited in the MARQ Museum (Alicante, Spain). The fieldwork excavation memory (with plans, stratigraphic sections, photographs and drawings) and all the original spreadsheets with the charcoal raw counts, faunal descriptions, land snail morphometrics are deposited at the Institut Català de Paleoecologia Humana i Evolució Social (Tarragona, Spain).

#### 3.2. Taphonomic analysis

To assess the land snail taphonomy and breakage patterns, we established four main fragmentation categories: 1. complete (uncrushed) snails, 2. partially crushed (up to the 50% of the whole shell) snails, 3. snail fragments (between 10–50% of the whole shell) and 4. snail debris or small snail fragments <10% of the whole shell. Small whorl fragments, of less than 3 mm, are very frequent and were not quantified since most of them were produced during the excavation and by post-depositional diagenetic processes.

Micro-DXR analyses were performed on a sample of two live specimens and four fossil land snails to compare the aragonitic-calcitic composition between present-day snails without burning traces and fossil shells recovered in the combustion areas. Analyses were made using a Bruker-AXS D8-Discover diffractometer equipped with a parallel incident beam (Göbel mirror), vertical θ-θ goniometer, XYZ motorised stage and a GADDS (General Area Diffraction System) in the Servei de Recursos Científics i Tècnics (SRCT) at Universitat Rovira i Virgili (URV) in Tarragona.

#### 3.3. Biometric analysis

Biometric analysis based on maximum height and diameter measures of all the complete specimens was undertaken to investigate the age selection patterns of land snail accumulations. All measurements were obtained using digital callipers and statistically analysed with the R software package [34]. In addition, to estimate the age of the archaeological assemblages, we compared the aforementioned measurements with those obtained from five different populations of modern specimens born and raised in the laboratory [35].

## Results

### 1. Taxonomic analysis and quantification

**Figure 4:**
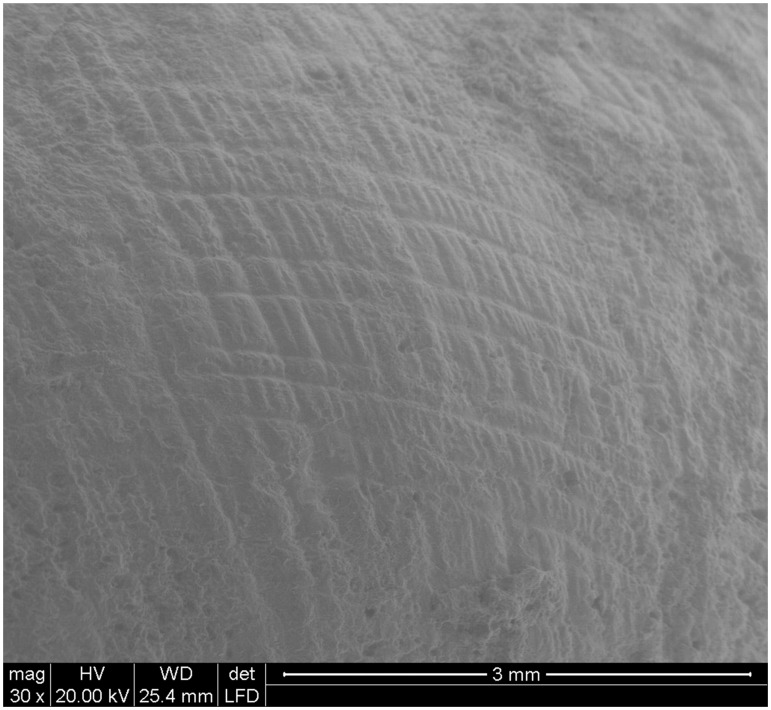
SEM microscope image showing the characteristic reticular sculpture of the genus *Iberus* shells.

Taxonomic identification of land snails, based on macroscopic and microscopic analyses, revealed that *Iberus alonensis* is the only species of land snail in the Upper Palaeolithic levels of the site. As SEM images clearly show (Figure 4), the shell surface displays a clear diagnostic reticular sculpture that is characteristic [36] of *I. alonensis*. That is the only species of the genus *Iberus* living in this area. It inhabits limestone Mediterranean environments with different vegetation formations, from open forests of pine and oak to xerophytic bushes [31]–[32]. Its life span ranges from ten to twelve years, and it displays two reproductive cycles during spring and fall.

Depending on the substrate, it estivates and hibernates buried or hidden in crevices. The occurrence of *I. alonensis* land snails has been reported, with very different frequencies, at several Late Pleistocene and Early Holocene archaeological sites in the southern and eastern Iberian Peninsula, where it has been interpreted as food remains [37]–[41].

Table 5 details the quantification of *I. alonensis* shells found in the Palaeolithic levels of Cova de la Barriada along with the excavated volume of the archaeological deposits. Almost 73% come from Level A and combustion structure BM, even though *I. alonensis* concentrations are also found in Levels B and C. If the shell quantitative values are normalised by volume of excavated sediment (NISP and MNI/m^3^), the picture that emerges is completely different. The combustion structure BM yields significantly much higher NISP and MNI values than Levels A, B, and C, whereas the previous quantitative differences between Levels A, B and C are less significant now.

**Table 5:** *Iberus alonensis* accumulations in the test pit 1 of Cova de la Barriada.

	Volume	NISP		MNI		NISP/m^3^	MNI/m^3^
	m	n	%	n	%	n	n
Level A	0.1415	733	49.39	418	50.24	5180	2954
BM	0.0085	356	23.99	180	21.63	41882	21176
Level B	0.0907	229	15.43	133	15.99	2240	1466
Level C	0.0946	166	11.19	101	12.14	1754	1067
Total	0.3353	1484	100	832	100	51056	2663

**Figure 5:**
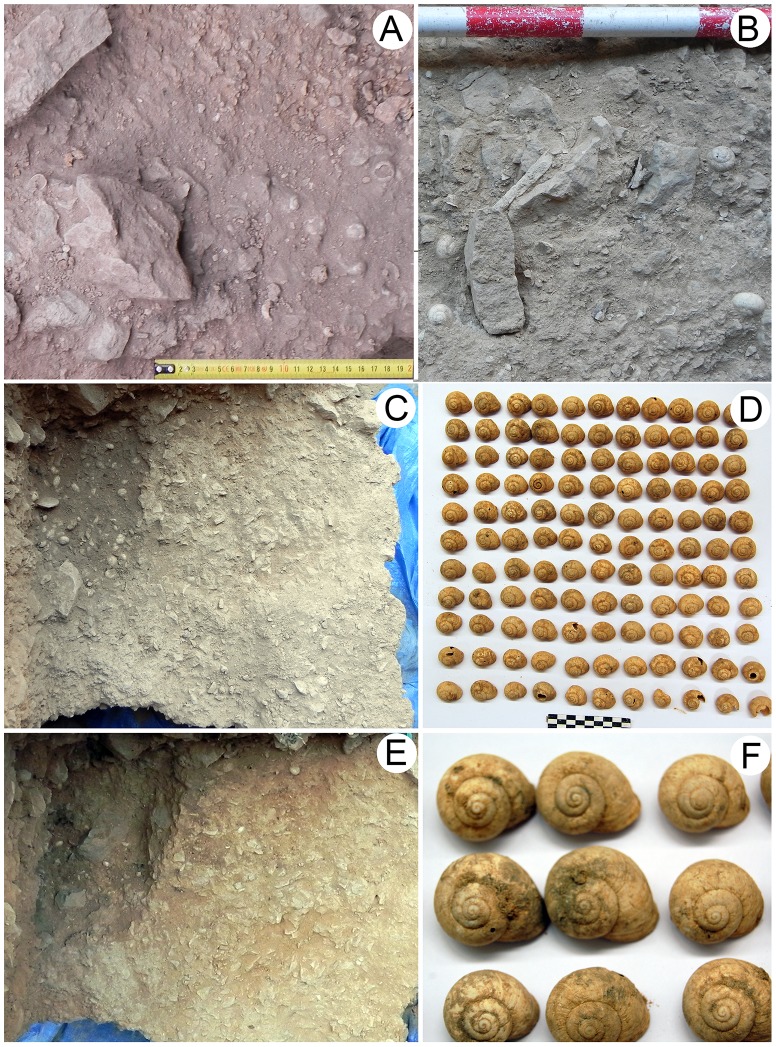
Cultural accumulations of *I. alonensis* land snails. (A) Spatial association of land snails to carbonaceous sediments and fire-cracked stones of combustion structure EC-1, (B) spatial association of land snails, bone fragments and lithic artifacts of level C, (C) combustion structure BM containing land snails and homogenous carbonaceous sediments; (D) land snails found into the combustion structure BM, (E) morphology of the combustion structure BM once excavated (maximum length 41 cm), (F) burning traces of *I. alonensis* found into the filling sediment of combustion structure BM.

According to the higher density values of *I. alonensis* shells, as well as the feature morphology and the anthracological record, the BM hearth structure can be interpreted as a specific-purpose cooking pit for roasting land snails. In contrast, the density values found at Levels A, B and C suggest a different accumulation pattern as waste around combustion and habitation areas (Figure 5).

### 2. Taphonomic results

Table 6 presents the breakage-size categories of *I. alonensis* land snails grouped by levels and reported as absolute and relative values regarding the NISP. Whole specimens and fragments preserving between 10–50% of the shell represent the most common classes, followed by fragments smaller than the 10%. Fragments larger than the 50% of the shell are the less common category in all the levels except for level C.

**Table 6:** Fragment conservation cathegories of *I. alonensis* land snails.

	Level A		BM		Level B		Level C	
	n	%	n	%	n	%	n	%
Whole	302	41.2	112	31.46	67	29.26	42	25.3
>50%	82	11.19	18	5.06	27	11.79	45	27.11
10–50%	164	22.37	157	44.1	80	34.93	50	30.12
<10%	181	24.69	69	19.38	55	24.02	29	17.47
Total	733	100	356	100	229	100	166	100

The high percentage of whole specimens and its mono-specific representation pattern is common in cultural accumulations and very rare in those land snail concentrations produced by small carnivores and birds [42]. Several studies have reported different patterns of shell damage produced by birds and mammals. Allen [43] reports a worldwide list of 67 species of birds eating land snails. However, just few works have provided detailed and comprehensive descriptions of the resulting shell breakage patterns. Turdidae birds -mainly the blackbird and the song thrush- produce marks of strikes on the left part of the last whorl [44]. In the case of the thrush song, as a result of using a stone anvil on which to break open the shells held by the peristome [42]. Other bird families with descriptions about land snails shell breakage patterns are the wekas (*Galliralus australis*) and the parrots[45]. Even though both species were not present in Europe during the Late Pleistocene, we can assume similar or close-related patterns of damage produced by other potential bird species as a result of the mechanical breakage of shells using the beak. The wekas produces pecking marks in the spire and early whorls as well as the base of a shell, making a hole through the centre [45]. On the other hand, parrots leave pairs of vertical scratches around the side of a shell and, also, can produce the removal of the early spirals [45].

In contrast, the number mammal species displaying predatory behaviour on land snails is considerably lower, 29 species[43]. Most of the literature about shell damage patterns mainly focuses on the feral pig, the badger (*Meles meles*), the europeam hedgehog (*Erinaceus europaeus*), the mice (*Apodemus sylvaticus*) and the ship rat (*Rattus rattus*). These species produce different patterns of damage depending on whether they eat the whole shell or they bite and/or gnaw part of the shell for accessing to flesh. Feral pigs exhibit a characteristic damage around the shell periphery and the flattened halves of the shells, often associated to teeth marks impressed as shatter points [45]. It is also reported the consumption of whole snails amongst feral pigs, a practice which leaves small fragments of shells in the faeces. On the other hand, badgers and hedgehogs produce a significant destruction of land snail shells in small fragments, with a differential preservation of the columnella [42].

Rodents such as the mice or rat break land snail shells in different ways. Because his small jaw size and mouth gape, the mice uses to concentrate his predatory activity on small or medium size land snails, mainly producing tooth bites on the aperture [44]. In contrast, the ship rat gnaws through the side of the shell and the inner whorls of the helicid snails [45], and can produce very distinctive damage patterns as the cracking of the lower part of the shell without affecting the lip or the proto-conche [46].

The shell damage produced by the above mentioned land snail predators is different from that observed in the archaeological specimens found at the Gravettian levels of Cova de la Barriada. First, it should be noted the high percentage of complete specimens, a fragmentation category seldom found in land snail assemblages produced by birds, insectivore mammals and rodents. Second, some big fragments (>50%) have fractures on the aperture, a pattern that can be created by small land snail predators such as the mice. However, small fractures -including those found on the aperture- can also be attributed to human manipulation during the land snail consumption, post-depositional damage caused by trampling and, even, the excavation process. Our next land snail fragmentation categories – medium size (10–50%) and small size (<10%)- overlaps with the size distributions produced by different avian and mammal predators which intensively break the land snail shells such as the Turdidae birds and, especially, the European hedgehog. However, the sum of both fragmentation categories found in the combustion structure BM, whose anthropic origin casts no doubts, reaches the 64%. Thus, medium and small size fragments can also be found in archaeological contexts completely produced by humans and affected by non-predatory post-depositional breakage such as trampling and/or sediment compaction. It is interesting to note that those highly diagnostic fractures produced by rats described by Moreno-Rueda [46] are absent in the archaeological assemblage presented here.

The taxonomic composition of land snail accumulations is an important variable to differenciate its anthropic or natural origin. Badgers tend to produce low-density accumulations of land snails with different taxonomic representation and a very diverse fragmentation pattern [42]. In contrast, land snail accumulations produced by Turdidae birds are quantitatively more abundant, displaying a narrow taxonomic selection (one or two species) and a very distinctive breakage pattern in the apex and the whorls, with no conservation of complete individuals [42]. The mono-specific composition of the land snail assemblage is a consistent argument against the natural accumulations produced by non-human predators. Different studies have shown that predation produced by birds and mammals focus on a restricted number of species, with a marked selection of birds toward large land snails [43], [44]. However, the taxonomic composition found in birds and mammal accumulations is not mono-specific, as supported by the quantitative evidence provided by Rosin on birds and mouses [44] and Faus on rat nests [47].

Finally, in the Iberian Peninsula, land snails are found in low-density accumulations in red fox lairs [48]. However, the origin of such accumulations is due to thanatocoenoses processes according to the documented high taxonomic and biometric diversity. As we will see in the next section, the biometric analysis of *I. alonensis* shells from Cova de la Barriada does not support a similar accumulation process to explain the presence of land snail at the site.

The relative frequencies of whole shells found throughout the archaeological sequence display a clear decreasing pattern from top to bottom, suggesting the occurrence of post-depositional mechanical breakage due to sediment compaction.

**Figure 6:**
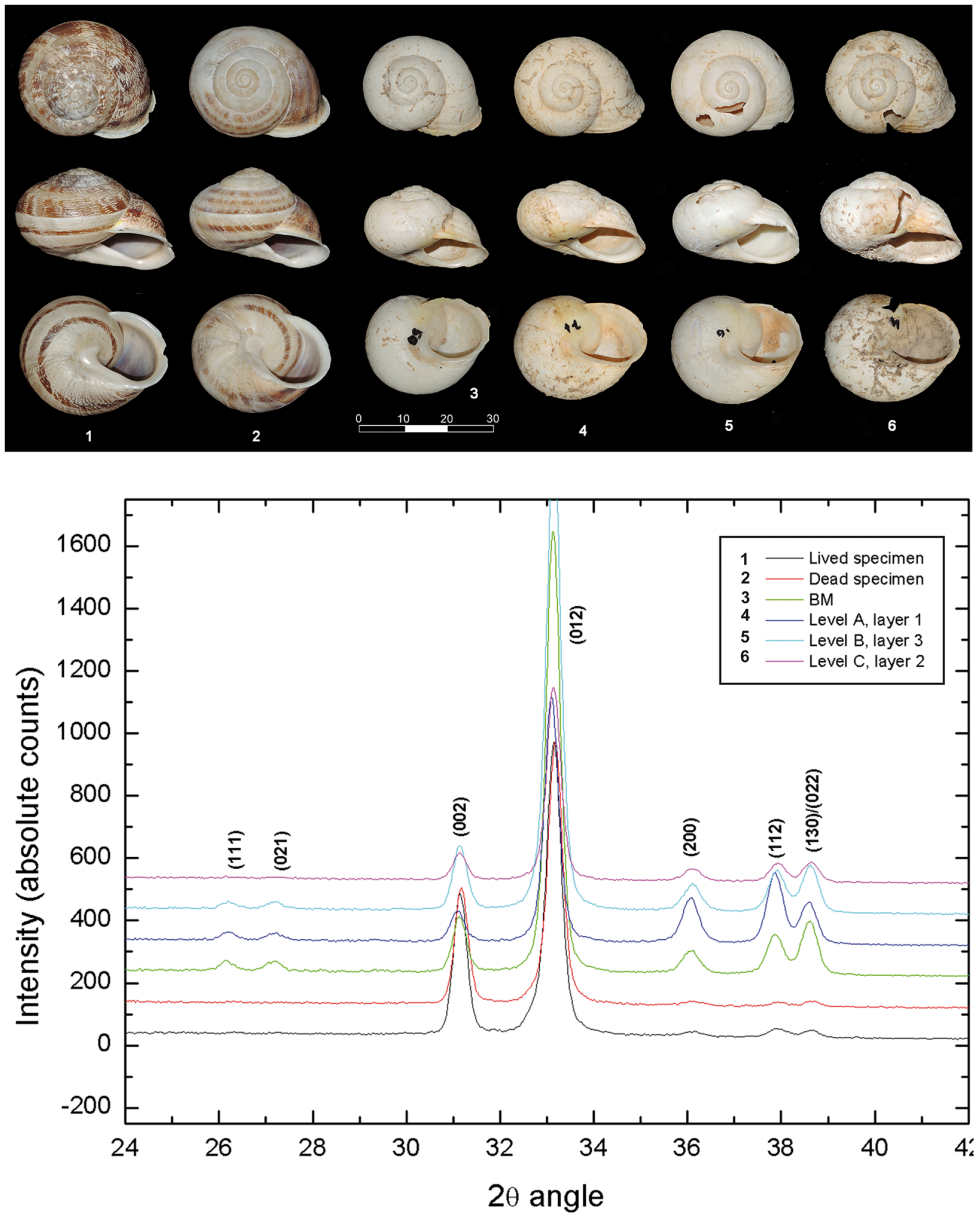
Up: X-ray diffractograms of modern (1–2) and fossil (4–6) *I. alonensis* shells. Difractograms have been moved vertically to avoid overlapped data. Vertical labels indicate the Miller indexes for aragonite diffraction peaks.

The results of the X-Ray diffraction analysis for each sample are shown in Figure 6. All samples presented aragonite (CaCO_3_) as the only mineral phase. The experimental relative intensities for aragonite do not match with the expected ones (when compared with the pattern 41–1475 of the ICDD database) because of the preferred orientation of aragonite crystals in the shell, which is very common in biogenic structures. According to J.E. Parker et al. [49], the samples analysed did not reach a temperature over ≈375°C, where the irreversible transformation of aragonite-calcite is expected to start in biogenic structures. However, the samples analysed presented some changes on the texture of aragonite that can be seen from the relative intensities of the peaks in the diffractograms. For samples BM, Level A and Level B, the peaks (111) and (021) are well detected, whereas for the other samples, such peaks have almost no intensity. It is well known that the annealing process introduces changes in the texture of bulk materials together with crystal phase transformation [50]–[51]. Unfortunately, no information has been found on how the aragonite texture in the snail shell changes as a function of the annealing treatment.

### 3. Biometric analysis

**Figure 7:**
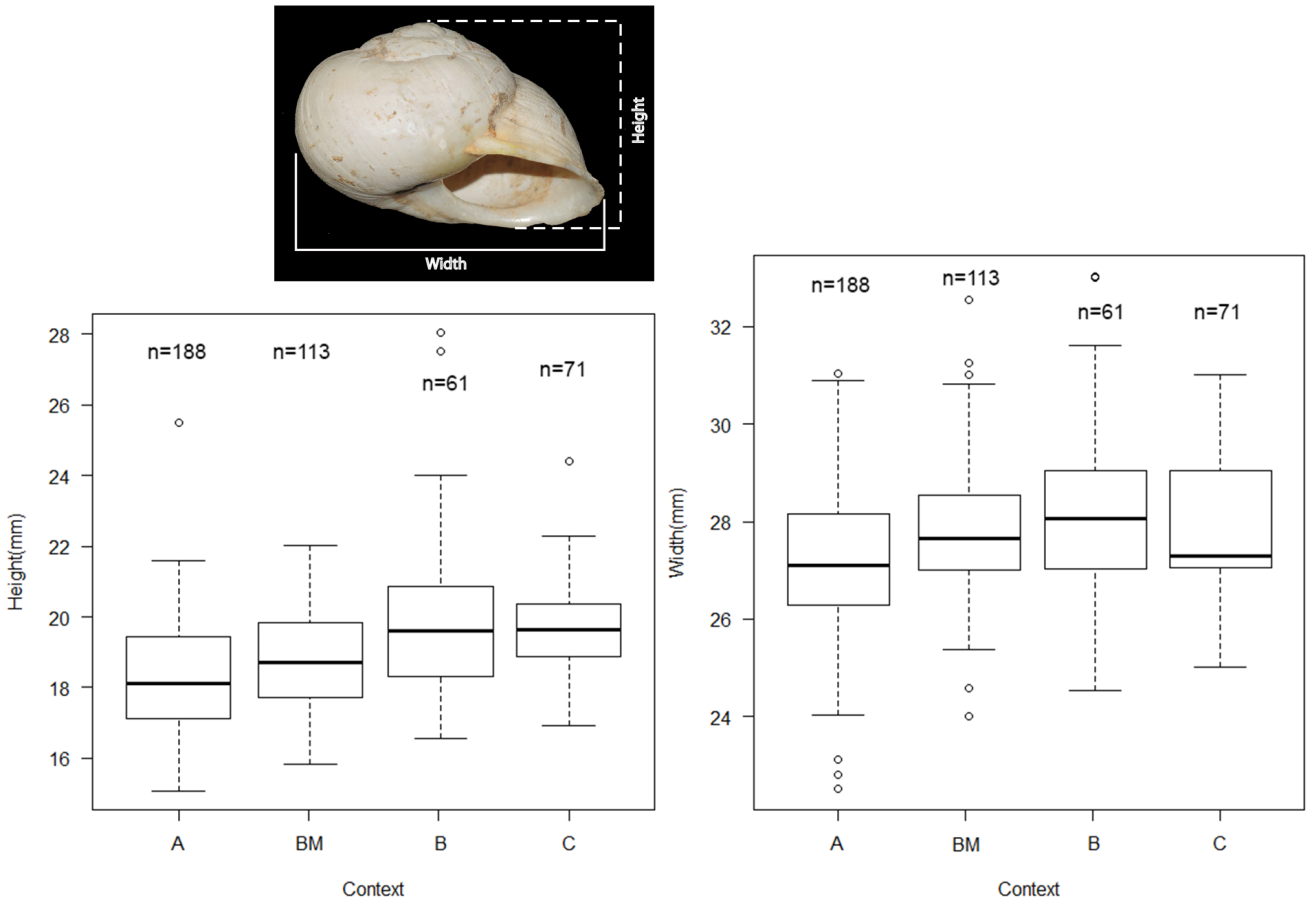
Boxplot of height and width measurementes of *I. alonensis* shells from Cova de la Barriada.

Land snail shell maximum height and width measurements were recorded using a digital calliper. The descriptive statistics of the width and height measurements for *I. alonensis* shells are reported in Table 7 and Figure 7 for the different levels and combustion structure BM: 432 shells were suitable for analysis, providing a mean value of 27.62 (±1.48) mm width and 18.79 (±1.67) height for all the specimens. The skewness values of all the snails indicate a closely normal distribution shape for the widths and a slightly skewed distribution of heights. The variance and standard deviation values indicate a narrow range of land snail widths (Std = 1.48), which is consistent with with shell size distribution recorded in archaeological records [52]–[55]. The higher degree of variation found in the skewness and standard deviation values of heights (Std = 1.67) is partially explained by the outliers found in Level B. However, if these outliers are removed, the variance and standard deviations values are much closer to those found in the other levels.

**Table 7:** *I. alonensis* sample and descriptive statistics of width and height measuruments through time.

	Width (mm)					Height (mm)				
	A	BM	B	C	Total	A	BM	B	C	Total
n	188	113	61	70	432	188	113	61	70	432
Mean	27.29	27.77	28.21	27.92	27.62	18.37	18.7	19.83	19.58	18.79
Median	27.11	27.66	28.06	27.29	27.43	18.12	18.71	19.61	19.63	18.72
Maximum	31.03	32.55	33.03	31.02	33.03	25.5	22.02	28.05	24.40	28.05
Minimum	22.52	24.01	24.53	25.02	22.52	15.07	15.84	16.56	16.93	15.07
Variance	2.01	2.81	1.89	1.84	2.19	2.23	1.77	4.6	2.47	2.79
Std	1.41	1.37	1.67	1.35	1.48	1.49	1.33	2.14	1.57	1.67
Skewness	−0.23	0.53	0.58	0.3	0.21	0.68	0.08	1.57	0.47	1.07
Kurtosis	3.58	3.96	3.75	2.35	4.01	4.31	2.44	6.91	3.7	6.58
1st Quartile	26.28	27.01	27.02	27.06	26.82	17.12	17.73	18.31	18.87	17.55
3rd Quartile	28.16	28.54	29.04	29.04	28.52	19.44	19.83	20.82	20.36	19.92

**Figure 8:**
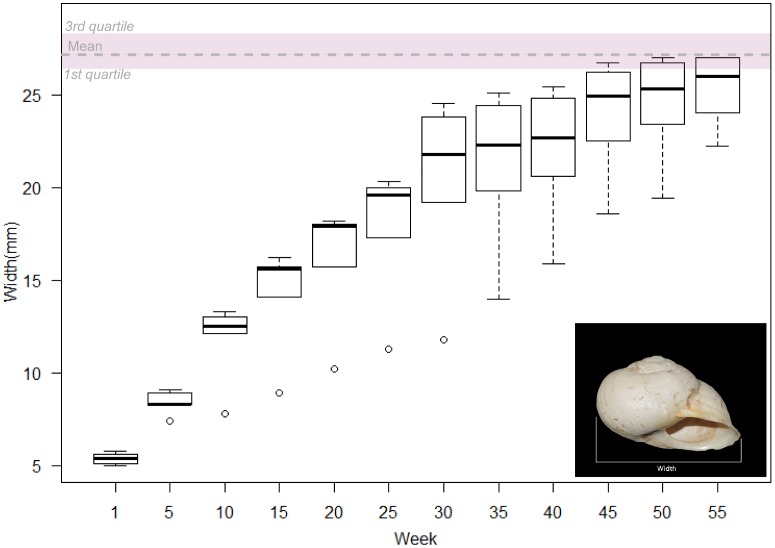
Boxplot of width measurements from 5 modern reference populations of *I. alonensis* during their first year of live (raw data obtained from [36]. The velvet band represents the interquartile range of the archaeological specimens found at the Gravettian levels of Cova de la Barriada.

The minimal variation in width and height values makes possible a tentative essay of age selection patterns on the basis of modern data of shell growth as it has been demonstrated that shell width and land snail age are positively correlated [35]. Figure 8 represents the width measures of five different populations of land snails whose progenitors were harvested at different localities in southeastern Spain, although reproduced and raised in the laboratory under controlled feeding and temperature conditions. Though the ideal comparative scenario would be that of using growth rates from wild specimens, such a procedure is impossible to achieve given the current-day overharvesting of *I. alonensis* and/or their replacement from many habitats by other gastropods. The use of different populations of individuals raised in the laboratory allows us to establish a first comparative analysis between controlled growth rates of live size variation of archaeological specimens.

The x-axis is a five-week temporal series within the first year of life, whereas the boxplots record the mean and the distribution values of width measurements obtained from the five modern reference populations, each one of them composed of 10 individuals. The pink band represents the interquartile range of the archaeological specimens and the dashed line the mean value. The distribution of width values of the archaeological specimens is significantly different from those individuals younger than 45 weeks found in the modern reference collection. On the other hand, individuals of 45 to 55 weeks age display a partial overlap with the width values exhibited by the fossil specimens, even though the mean values are consistently different. Despite the fact that the relationship between age and shell size of modern and Late Pleistocene *I. alonensis* might not be exactly the same, given the distinctive environmental conditions at the end of MIS 3 [56], both observations, the narrow range of the fossil widths and the significant differences regarding the shell size of land snails younger than 45 weeks, suggest a strong selection pattern where immature individuals were ruled out. In addition, the fragmentation pattern observed in the archaeological specimens supports this interpretation. The lack of juvenile (and more fragile) shells seems not to be explained by sediment compaction because, in each level, the “whole shell” category is more frequent than the small fragments <10%.

## Discussion

### 1. Land snail accumulations and cultural selection patterns

Anthropogenic accumulations of large snails dated during the Gravettian period (31.3–26.9 kyr cal BP) have been reported on the basis of taxonomic, taphonomic and biometric data and their association with occupational features, lithic and faunal assemblages. Land snails found in the Gravettian levels of Cova de la Barriada reveal the existence of mono-specific gathering strategies focused on large pulmonate gastropods during the Early Upper Palaeolithic. Taphonomic analyses indicate significant differences between the breakage patterns documented for the Gravettian levels of Cova de la Barriada and those produced by other land snail accumulators such as birds, hedgehogs, badgers, mices and rats. The most significant difference is the high representation of complete shells. Such a pattern is very uncommon in the shell remains left by other land snail predators, which systematically destroy part of the shell spiral, the sides or the aperture for gaining access to flesh. As it has been observed before, mechanical breakage due to sedimentary compaction was responsible for some land-snail fractures, as reported with other zooarchaeological remains. However, the high frequencies of complete specimens and their clear spatial association with combustion structures advocate for the cultural origin of the land snail accumulations as food remains. Furthermore, the micro-DXR analysis indicates differences in the aragonitic composition between live and fossil specimens, suggesting that the archaeological samples were heated under controlled conditions (below 375°C) given the null conversion of aragonite into calcite. Recent experiments on the prehistoric cooking process of the large edible land snails *Helix* sp. indicate that roasting land snails on embers between 5-8 minutes is the most plausible technique documented at Pupicina cave, on the eastern Adriatic area, leaving barely visible traces of burning [57]. In addition, our analytical protocol using DXR indicates that because of post-depositional bleaching, fossil specimens without clear macroscopic evidence of burning were heated as well, given the presence of different aragonitic signals not found in modern specimens. Furthermore, charcoal data from the combustion structure BM, which delivered 112 complete land snails, provides fresh evidence about the fuel employed in the cooking process of the land snails: *Pinus nigra/sylvestris* and juniper charcoals, most of them between 2 and 15 timber rings. The structure BM is different from other examples of combustion structures used to cook land snails dated to the Capsian in Algeria. The Capsian examples consist on hearth pits delimited by vertical stone slabs and filled by two layers of heated stones between which the land snails are boiled by heating radiation [58].

On the other hand, the biometric analysis of *I. alonensis* shell widths and heights indicates a narrow selection pattern of land snail sizes. The comparative analysis between the archaeological specimens and modern land snails raised in the laboratory suggest that most of the gathered individuals were older than one year, and those younger than 45 weeks were not gathered at all. Such a strong age selection pattern suggest sustainable exploitation of *I. alonensis* based on knowledge of its reproductive cycle.

### 2. Cultural and economic implications of land snail consumption during the Early Upper Palaeolithic

**Figure 9:**
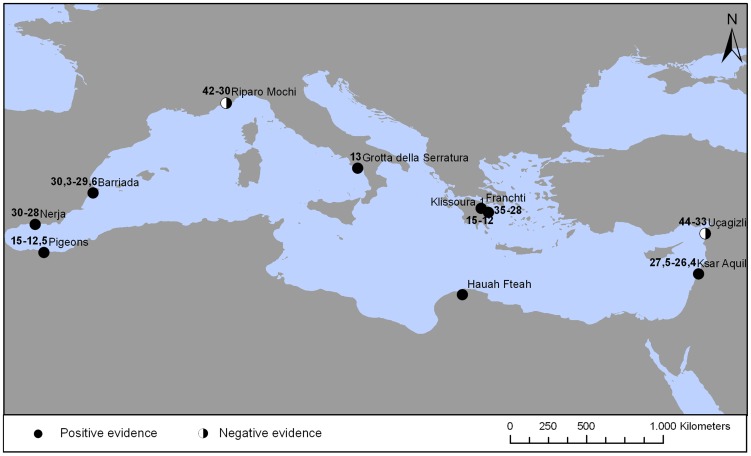
Map of Upper Palaeolithic sites of the Mediterranean basin displaying the earliest occurrence of land snails as food remains. Bold numbers indicate the calibrated chronology in ka yrs (see also table 8). The black & white symbols denote Early Upper Palaeolithic sites with a consistent pattern of small prey exploitation including shellfish and lacking evidence of land snail consumption.

The body of archaeological evidence about the consumption of edible land snails by humans prior to the Late Glacial Maximum is scarce, and its interpretation as food remains is rarely accepted for such an old chronology [17]–[18]. In Table 8 and Figure 9, we present a list of Upper Palaeolithic contexts recording the first cite about the use of terrestrial gastropods as a food resource, with the corrected chronology and taxonomic identification.

**Table 8:** Earliest evidence of systematic conssumption of land snails.

	Site/Level	Culture	ka yrs cal BP	Species	Reference
**East Africa**	Mumba/Vupper	Later Stone Age	49.1±4.3*	*Achatina* sp.***	[59]–[61]
	Mumba/III lower	Later Stone Age	36.8±3.4*	*Achatina* sp.***	[59]–[61]
**Levant**	Ksar 'Aqil/3b	Late Upper Paleolithic	27.5–26.4	undetermined	[17], [62]–[63]
**Greece**	Klissoura 1/IIIc	Upper Aurignacian	35–28**	*Helix figulina*	[65]–[66]
	Franchthi/S2-T3	Epigravettian	15–12	*Helix figulina*	[67]
**Italy**	G. della Serratura/8G	Late Epigravettian	14.1–13.7	*Helix* cf. *ligata*	[52]
**North Africa**	Taforalt/grey series	Iberomaurisian	15–12.5	*Theba* sp., *Cornu aspersum, Otala punctata*	[80]
	Haua Fteah/XVI-XXV	Dabban	43–17	*Helix melanostoma*	[70]–[71]
**Iberia**	Nerja/IV	Gravettian	27.9–26.9	*Iberus alonensis*	[80]–[81]
	Barriada/C, B, A	Gravettian	31.3–26.7	*Iberus alonensis*	This study

*Optically stimulated luminiscence dating of quartz and feldespar grains.

**dating estimates inferred by the chronology from the layers above and below.

***Lack of detailed quantitative and evidence.

The first acknowledged evidence of land snail consumption by humans [17] comes from the Mumba-Höle site (Tanzania), a reference archaeological sequence of East Africa. Melhman [59] reported the existence of middens of the large land snail *Achatina* sp. in both beds III-lower and V dated to 31070±500. It is worth noting that such accumulations occurred in association with rich artefact assemblages, oyster shell beds and crescent microliths. Melhman interpreted the lithic industry of level V as a Middle Stone Age (MSA) and Later Stone Age (LSA) transitional industry. However, new excavations at this site and the production of new dates allow revision of the previous cultural interpretation of level V as a transitional industry to a full LSA lithic assemblage [60]. Recent contributions on the chronology of the archaeological sequence[61], through optically stimulated luminescence dating of quartz and feldspar grains, yielded however, older ages for levels III inf (36.8±3.4 kyrs) and V upper (49.1±4.3 kyrs). The specific study of the land snail assemblages remains unpublished.

In the Levant, the Ksar 'Aqil (Beirut) site yielded land-snail accumulations in Level 3b of Tixier's excavations [62], dated to between 22,000 and 23,000 BP uncalibrated [63]. It is difficult to maintain, with the current data, the older use of land snails as food resource in this region. For instance, further north, at Üçagizli Cave (Hatay, Turkey), a well-documented archaeological sequence of the Initial Upper Palaeolithic and the Ahmarian dated to 44–33 kyrs cal BP [64], there were found several land snail species, including *Pomatias elegans, Xeropicta* sp. and *Helix* cf. *engaddensis*, which accumulated through natural processes. Despite the significant representation of the small game at the site, with several species of fish, leporids, birds, tortoises and shellfish, there is no evidence for the use of pulmonate gastropods as food.

In the Argolid, Franchthi Cave and Klissoura Cave 1 have been thoroughly investigated, yielding a different chronological pattern for the consumption of *Helix figulina*, the main edible land snail species documented in the Late Pleistocene and Early Holocene archaeological deposits of this region. At Klissoura Cave 1, *Helix figulina* land snails occur from the Upper Palaeolithic to the Mesolithic. The shells found in the Uluzzian and the Early Aurignacian units (Layers V and IV) lack evidence of human modification [65]. In contrast, land snails become moderately abundant from the Upper Aurignacian (Level IIIc), with evidence of high percentages of broken lips, interpreted as evidence of human consumption, growing exponentially up to the Mesolithic (Layers 3–5). No radiocarbon information is available for Layer IIIc, whose chronology spans between the dates of the underlying Middle Aurignacian units (IIIe-IIIg), dated to 35–36 kyrs cal BP, and the overlying Upper Palaeolithic levels (III″), dated to c. 28 kyrs cal BP [66]. The antiquity of land snail exploitation documented at this site is not mirrored at the neighbouring Franchthi cave, where food debris of *Helix figulina* occurs much later, in the Epigravettian units (S2-T3) dated to 15–12 kyr cal BP [67].

At Haua Fteah cave in Cyrenaica, land snail accumulations interpreted as food debris were reported in the pre-Aurignacian, Iberomaurisian, Capsian and Neolithic layers of McBurney excavations [68]–[69]. New excavations [70]–[71] have documented land snails throughout the whole archaeological sequence. Detailed quantitative information about land snail occurrence is available for the uppermost part of the sequence, Layers III-X, from the Historic period up to the Libyco-Capsian [72]. At these layers, as well as in the neighbour site of Hagfet al-Gama, the shells of *Helix melanostoma, Rumina decollata*, and *Trochoidea cretica* are thought to represent food debris. For the earlier prehistoric occupations at Hauah Fteah, preliminary data indicates land snails were used as food resources during the Oranian (c. 14–10 ka cal BP) to a lesser extent than during the Lybico-Capsian, whereas during the Dabban period (c. 40–15 ka cal BP) their use seems occasional, and no food molluscs are documented during the Mousterian layers [70]. Future works at this site are needed to elucidate the beginning of the systematic and continuous exploitation of land snails along the Dabban occupations, whose radiocarbon record covers a chronological span of more than 25,000 calendric years [71].

In the Balkans and Italy, cultural accumulations of land snails have been reported from the Late glacial and the Early Holocene onwards [52], [73]–[76] but not during earlier periods. For instance, Level G of Riparo Mochi (Aurignacian) yielded land snail accumulations (not taxonomically determined), but on the basis of the taphonomic evidence such as perforation marks, the lack of burning traces and spatial distribution, Stiner [3] suggested they were accumulated by avian and small mammal predators. In southern Italy, Grotta della Serratura, a long cave sequence spanning the Gravettian, Evolved Epigravettian and Late Epigravettian occupations is paradigmatic. Despite the presence of marine and terrestrial molluscs throughout the sequence, the consumption of *Helix* cf. *ligata* is just documented in the Late Epigravettian layers [75]. In contrast, in Sicily, the pulmonate gastropod *Eobania vermiculata* is found throughout the archaeological sequence from Grotta d'Oriente, from the Late Upper Palaeolithic to the Late Mesolithic, even though there is no evidence of human collection for food consumption [77]. Other Late Glacial and Early Holocene Italian sites such as Grotta della Madonna a Praia a Mare and Grotta del Mezzogiorno have provided abundant evidence of *Helix* cf. *ligata* land snail shells [78]–[79]. However, its interpretation as food remains is still inconclusive because the malacological assemblages come from old excavations.

In the Magreb, at Grotte des Pigeons, recent malacological studies [80] demonstrate an intensive exploitation of land snails associated to the grey series of Iberomaurisian affiliation dated to 15–12.5 kyrs cal BP. In contrast, the land snails found in the underlying yellow series were not the result of human agency. At the same site, a few large specimens of *Otala punctata* were reported in the Aterian levels, associated with thin ash lenses dating to around 80,000 BP, suggesting “a very small scale exploitation”[80∶13] even though no detailed description and quantification are available for such an older occurrence.

Finally, at Cueva de Nerja (south Iberian Peninsula), accumulations of *Iberus alonensis* are found throughout the archaeological sequence, from the Gravettian to the Chalcolithic. The oldest occurrence is documented in Levels 13 and 11 of the Vestíbulo chamber, where continental gastropods are dominant during the Gravettian and during the subsequent Solutrean occupations [81]–[82].

According to this review, the exploitation of edible land snails is a subsistence activity restricted to anatomically modern humans post-dating the Middle to Upper Palaeolithic transition, geographically restricted to some of the warmest European regions during the final stages of the MIS 3. During the beginning of the Early Upper Palaeolithic, edible land snails appear have accumulated in archaeological deposits through natural processes and/or non-human predators in the Eastern and Central Mediterranean as well as the Magreb. Our study reveals, however, that the exploitation of large edible land snails in the Iberian Mediterranean region is clear from the beginning of the Gravettian techno-complex c. 31.3–26.6 kyr cal BP onwards as indicated by the Solutrean and Magdalenian levels of Nerja cave. While the information about the taxonomic composition, taphonomy and biometrics of some key sites for the Early Upper Palaeolithic, Haua Fteah and Ksar 'Aqil, is rather limited, the remaining datasets clearly point to a later occurrence, associated with posterior Epigravettian or Iberomaurisian contexts after the Late Glacial maximum as documented in Grotte des Pigeons, Grotta della Serratura or Franchthi Cave. The main question posed by the Gravettian accumulations of land snails found at Barriada and Nerja caves addresses the origin of such a new economic behaviour, not reported in the Iberian Peninsula during the Middle Palaeolithic nor the Aurignacian periods.

Cova de la Barriada suggests that the exploitation of *I. alonensis* land snails by humans entailed a rigid mono-specific gathering strategy and a clear age-size selection pattern, culturally transmitted and maintained through generations. Their incorporation into the human subsistence systems during the beginning of the Gravettian could be interpreted as a regionally specific component of a broader range of complementary foraging and gathering activities focused on small prey [29], entailing a marked gender or age-related division of labour operating in a context of population growth [83].

Considering both the exploitation of marine molluscs during the Middle Palaeolithic and the lack of evidence on land snail consumption in Early Upper Palaeolithic contexts prior to the 31 ka yrs cal BP, there are no arguments to interpret the incorporation of land snails into the human diet in terms of different cognitive capabilities between Neanderthals and Anatomic Modern Humans. Rather, as the eastern and southern Iberian record indicates, the appearance of this new subsistence activity was coeval with other demographically driven transformations such as the significant increase of the number of sites [84]–[86], and beginning of the production of portable art [87]–[88].
